# Genetic Diversity and Divergence between Southern Japonica and Northern Japonica Rice Varieties in China

**DOI:** 10.3390/genes15091182

**Published:** 2024-09-09

**Authors:** Zhiqiang Yan, Ruyue Deng, Huihui Tang, Susong Zhu

**Affiliations:** Guizhou Provincial Academy of Agricultural Sciences, Guiyang 550000, China; yanzhiqiang1118@163.com (Z.Y.); 17311992182@163.com (R.D.); tanghuihui0609@163.com (H.T.)

**Keywords:** southern and northern japonica varieties, genetic diversity, genetic divergence, phenotype

## Abstract

Given the notable ecological and breeding disparities between southern and northern rice regions, delving into the genetic diversity and divergence between southern and northern japonica rice contributes to enhancing the genetic pool for japonica rice breeding. In this study, we analyzed 90 southern and 51 northern japonica rice varieties, focusing on nucleotide diversity (Pi), agronomic trait variations, population structure, genetic divergence, and a neutral test. For genetic diversity, the results demonstrated higher Pi in northern japonica rice varieties (NJRVs) on Chr2, Chr5, Chr6, Chr8, and Chr10, whereas in southern japonica rice varieties (SJRVs) on Chr7 and Chr9. In addition, SJRVs exhibited higher grain width and thickness, whereas NJRVs featured a higher grain aspect ratio, filled grain number, and grain number per panicle. Regarding genetic divergence, geographic differentiation existed between NJRVs and SJRVs, with Chr5 exhibiting numerous higher genetic differentiation windows, including cloned grain shape-controlling genes *RGA1* and *SFD5*, stemming from intensified selection pressure on SJRVs. In summary, SJRVs and NJRVs exhibited diversity differences and genetic differentiation. Hence, it was suggested to conduct genetic introgression between NJRVs and SJRVs to broaden the genetic basis of the local japonica rice germplasm. By exploiting their heterotic advantage, new japonica rice cultivars with superior comprehensive traits could be developed.

## 1. Introduction

In China, japonica rice accounts for 29.5% of the nation’s total rice area [1]. Its popularity increased in the market due to its excellent quality [2]. Japonica rice cultivation regions are split into southern and northern areas. The rice cultivation areas north of the Qinling Mountains-Huaihe River are categorized as the northern rice region, comprising Heilongjiang, Jilin, Liaoning, Tianjin, Shandong, and Xinjiang, etc., while those south of the line were designated the southern rice region, including Jiangsu, Zhejiang, Yunnan, Guizhou, Anhui, Shanghai, and Hubei etc. The northern rice region is characterized by higher latitudes, lower annual temperatures, scarce precipitation, and a dry climate, whereas the southern region exhibits opposite conditions [3,4]. Genetic diversity denotes the genetic variation within a population, while factors like geographic isolation and ecological differentiation result in genetic differences between populations, termed genetic divergence. Hence, it is crucial for japonica rice variety breeding to explore the genetic diversity of japonica rice in both the northern and southern region. Genetic diversity can be evaluated through phenotype [5,6], biochemical markers, and molecular markers [7]. Wang et al. analyzed the genetic diversity of japonica rice across different regions of Northeast Asia using simple sequence repeat (SSR) markers, indicating a low genetic diversity of japonica rice in Northeast Asia [8]. Liu et al. utilized SSR markers to analyze the genetic diversity of 233 japonica rice varieties from the northeastern region of China, revealing that Jilin province had the highest genetic diversity [2], and this result was in agreement with results of another study based on the Single Nucleotide Polymorphism (SNP) [9]. Gong et al. assessed the genetic diversity of 80 japonica rice varieties based on eight agronomic traits and clarified the comprehensive rankings [5]. Zhu et al. showed that high-quality northern japonica rice had lower pasting temperatures and better texture and that southern semi-glutinous japonica had higher breakdown values and higher viscosity [10]. Ma et al. showed that northern japonica had higher amylose and lower protein in contrast to southern japonica [1]. Gao et al. employed 8K SNP-Chips to characterize the genetic diversity of 137 parental lines of japonica hybrid rice, revealing that restorer lines exhibited greater diversity compared to sterility lines [11]. Wu et al. used kompetitive allele specific PCR (KASP) markers to analyze japonica rice varieties’ diversity and kinship, finding that Guizhou He was genetically close to japonica rice in Jiangsu [12]. The Fixation Index (*Fst*) is a pivotal genetic statistic that quantifies the degree of divergence between two populations, not only facilitating insights into their genetic diversity but also serving as a cornerstone for comprehending biodiversity and evolution [13,14]. A study comparing the *Fst* of temperate japonica cultivated rice and temperate japonica weedy rice revealed significant genetic differentiation in *OsGF14h* regulated flood adaptation between the two populations [15]. Comparisons of the *Fst* between *Oryza rufipogon* (*Or-ru*) and *O. indica*, as well as *Or-ru* and *O. japonica*, revealed that *TGW2*, which controls grain width and weight in rice, has undergone domestication selection in both *O. indica* and *O. japonica*, leading to a significant reduction in genetic diversity [16]. Although a great deal of research has been conducted on the genetic diversity of japonica rice from different regions, many studies often focused solely on evaluating the diversity of japonica rice germplasm within a specific region, exploring changes in genetic diversity over time and the homogenization among approved japonica rice varieties, while seldom delving into the comparison of diversity and genetic differentiation between southern and northern japonica rice, integrating both whole-genome sequence and phenotypic profiling [17,18,19,20,21,22,23,24,25,26,27]. This may limit opportunities for broadening the genetic foundation of japonica rice. This study employs 141 japonica rice samples from both southern and northern regions, comparing their phenotypic and molecular marker diversity. It analyzes highly genetically differentiated segments within the genomes of southern and northern japonica rice, investigating the presence of genes in these segments that impact agronomic traits. This research aids in facilitating genetic exchange between southern and northern japonica rice and supports the development of novel japonica rice varieties with superior comprehensive traits.

## 2. Materials and Methods

### 2.1. Plant Materials

In total, 141 japonica rice varieties were collected from 12 regions, with 90 from southern rice areas and 51 from northern rice areas. The number of varieties collected from each region was as follows: 20 from Yunnan (Tropical and Subtropical Zones), 7 from Guizhou (Subtropical Zone), 6 from Hubei (Subtropical Zone), 7 from Anhui (Warm Temperate and Subtropical Zones), 18 from Zhejiang (Subtropical Zone), 8 from Shanghai (Subtropical Zone), 24 from Jiangsu (Warm Temperate and Subtropical Zones), 2 from Shandong (Warm Temperate Zone), 2 from Tianjin (Warm Temperate Zone), 34 from Liaoning (Mid-Temperate and Warm Temperate Zones), 4 from Jilin (Mid-Temperate Zone), 9 from Heilongjiang (Cold Temperate and Mid-Temperate Zones) (Supplementary Table S1). Regarding regional selection, the three northeastern provinces (Liaoning, Jilin, Heilongjiang) excelled in japonica rice breeding in the north [28], while Jiangsu, Zhejiang, and Yunnan led in the south [29,30,31]. Consequently, these regions contributed a substantial portion of our japonica rice germplasm collection. Primarily, we focused on approved homozygous varieties or stable strains, without conducting further screening based on specific characteristics.

### 2.2. DNA Extraction, Library Construction and Sequencing

A total of 30 days after transplantation, the leaves of all japonica rice varieties were excised and immersed in liquid nitrogen, followed by storage in a −80 °C freezer. The total DNA of all samples was extracted using standard protocol with a DNA extraction kit (TIANGEN, DP305-03, Beijing, China). The detection of DNA samples mainly included three methods: (1) agarose gel electrophoresis to analyze the purity and integrity of DNA; (2) Nanodrop (Thermo Fisher, Waltham, MA, USA) to detect the purity of DNA (OD260/280 ratio); (3) Qubit 2.0 (Invitrogen, Singapore) for precise quantification of DNA concentration. Qualified DNA samples were randomly fragmented into 350 bp fragments by a Covaris crusher (Diagenode, B01080010, Antwerp, Belgium). The DNA fragments were then subjected to end repair, polyA tail addition, sequencing adapter ligation, purification, and Polymerase Chain Reaction (PCR) to complete the entire library preparation. The constructed library was sequenced using Illumina Novaseq™ 6000 (LC-Bio Technology Co., Ltd., Hangzhou, China), and the sequencing model was a 2 × 150 bp paired-end sequencing (PE150).

### 2.3. SNP Detection and Analysis

Prior to alignment, the low-quality reads were removed by fastp 0.20.0. For the alignment step, Burrows Wheeler Aligner 0.7.12 was utilized to align reads to reference genome Os-Nipponbare-Reference-IRGSP-1.0. As the initial post-alignment processing step, we utilized Picard tools 1.119 to identify and mark duplicate reads from the BAM file. In the subsequent step, local read realignment was performed to correct for potential alignment errors around InDels. To ensure the accuracy of the detection results, the HaplotypeCaller algorithm of GATK 4.0 (for local haplotype assembly) was then used for variant detection of SNPs. Each sample was individually processed to generate gVCFs, which were subsequently merged. Finally, after applying rigorous filters, we obtained the definitive set of variant sites.

### 2.4. Genetic Diversity Analysis

The 100 kb window-based nucleotide diversity (Pi) was analyzed using VCFtools 0.1.17 [32]. Data was visualized via GraphPad prism 9. Agronomic traits were determined through replicated trials conducted over a two-year period, spanning from 2020 to 2021 in Guiyang. Each variety was planted in two rows, and each row consisted of 20 individual plants. The inter-row spacing and inter-plant distance were 30 cm and 16 cm, respectively. At the maturity stage, three individual plants were taken to measure plant height, panicle length, grain length, grain width, grain thickness, filled grain number, grain number per panicle, and seed setting rate. A Student’s *t*-test was performed using SPSS 20.0.

### 2.5. Genetic Divergence Analysis

Kinship coefficients were estimated among individuals in our sample using Tassel 5. The generated kinship coefficients were translated into a kinship heat map in R (version 4.2.3) using the gplots package for easy visualization of the relationships. In an attempt to estimate the effective number of populations, an analysis of population structure was conducted based on an admixture 1.3.0 analysis with seven estimated ancestral populations (k), 10 iterations/runs for each k, and 100,000 repetitions [33]. The extent of admixture of the populations was illustrated with GraphPad prism 9, and inferred populations were colored differently for easy visualization. A 100 kb window-based Fixation Index (*FST*) and 500 kb window-based Tajima’s *D* were analyzed using VCFtools 0.1.17 [32]. Data was visualized via GraphPad prism 9.

## 3. Results

### 3.1. Genetic Diversity Analysis between SJRVs and NJRVs

**Figure 1:**
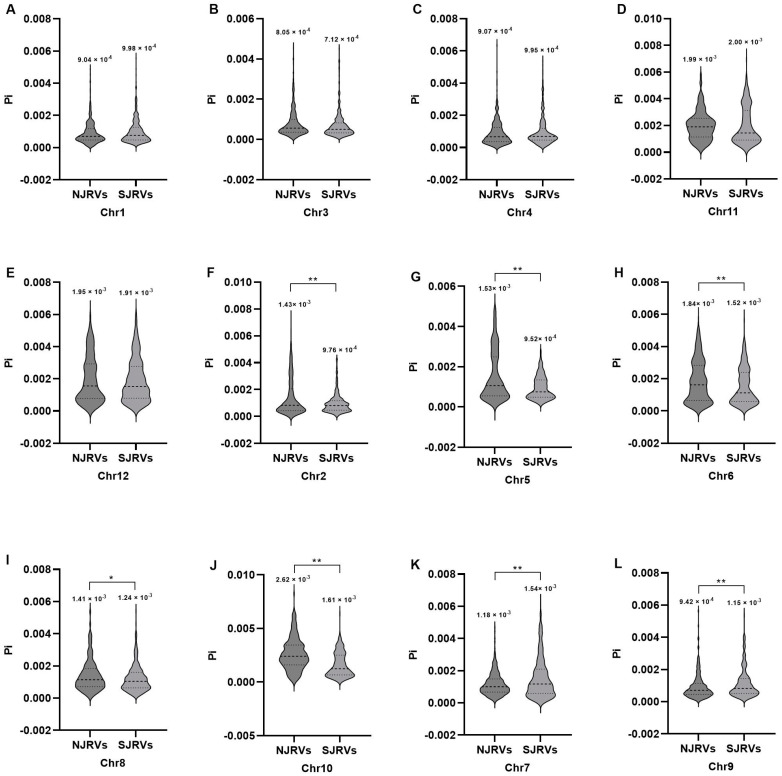
Analysis of nucleotide diversity (Pi) between SJRVs and NJRVs. (**A**–**L**) Nucleotide diversity of Chr1–Chr12, respectively. The y-axis represents 100 kb window-based Pi. The labelled values on the violin represents the mean value of Pi. Dotted dashes and short horizontal dashes represent quartiles and medians, respectively. Significant differences were determined using the Student’s *t*-test. “*”and “**” mean significant and extremely significant at the levels of 0.05 and 0.01, respectively.

The variance in Pi values exhibited by NJRVs and SJRVs was statistically insignificant across Chr1, Chr3, Chr4, Chr11, and Chr12 (Figure 1A–E). Across Chr2, Chr5, Chr6, Chr8, and Chr10, the Pi value was notably higher in NJRVs compared to SJRVs (Figure 1F–J). Conversely, on Chr7 and Chr9, the Pi value was significantly greater in SJRVs than in NJRVs (Figure 1K,L).

**Figure 2:**
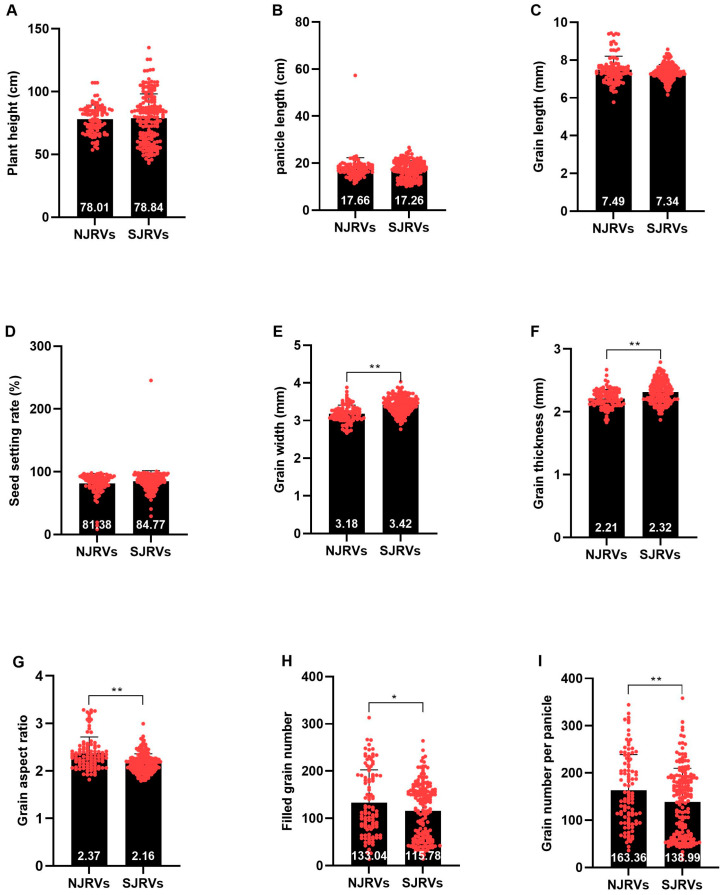
Analysis of variance for agronomic traits. (**A**) Plant height. (**B**) Panicle length. (**C**) Grain length. (**D**) Seed setting rate. (**E**) Grain width. (**F**) Grain thickness. (**G**) Grain aspect ratio. (**H**) Filled grain number. (**I**) Grain number per panicle. Significant differences were determined using a Student’s *t*-test. “*”and “**” mean significant and extremely significant at the levels of 0.05 and 0.01, respectively.

The differences in plant height, panicle length, grain length, and seed setting rate between NJRVs and SJRVs were not significant (Figure 2A–D). Grain width and thickness of NJRVs were very significantly smaller than those of SJRVs (Figure 2E,F), while the grain aspect ratio, filled grain number, and grain number per panicle were notably larger than those of SJRVs (Figure 2G–I).

### 3.2. Genetic Divergence Analysis between SJRVs and NJRVs

**Figure 3:**
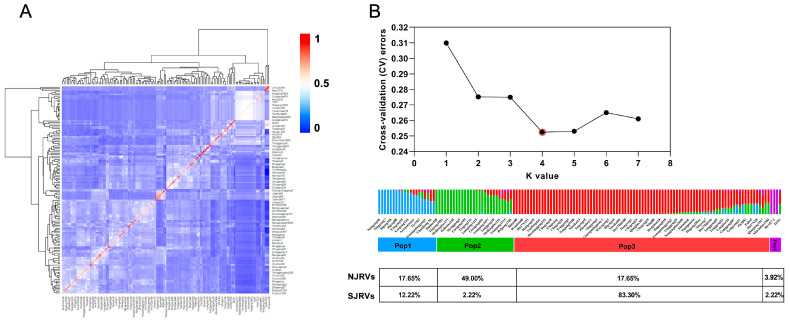
Kinship and population structure analyses. (**A**) Relationship between the samples. The color bar on the right side indicates coefficient of kinship. (**B**) Cross-validation and population structure. The red circle corresponds to the minimum CV value.

Kinship analyses showed low kinship coefficients among all samples, suggesting that no visible lineage differentiation occurred (Figure 3A). We determined the optimal number of clusters using the K value corresponding to the inflection point of the CV. The structure showed that the proportions of NJRVs in four subpopulations were 17.65%, 49.00%, 17.65%, and 3.92%, respectively, with the highest in Pop2, while those of SJRVs were 12.22%, 2.22%, 83.30%, and 2.22%, respectively, peaking in Pop3 (Figure 3B). Upon analysis, a distinct geographical differentiation was evident between SJRVs and NJRVs.

**Figure 4:**
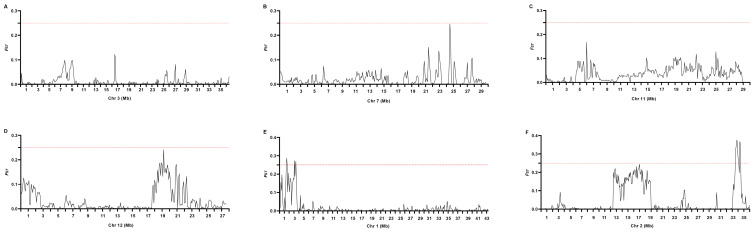

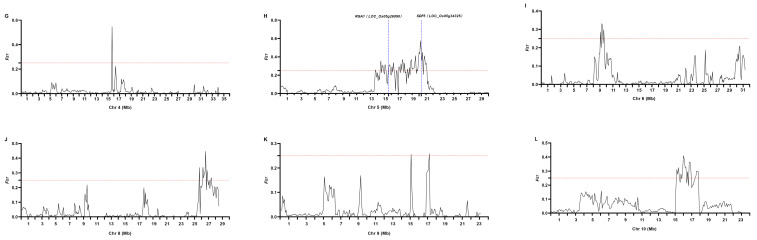
Analysis of Fixation Index (*F_ST_*) between SJRVs and NJRVs. (**A**–**L**) Analysis of Fixation Index (*F_ST_*) of Chr1–Chr12, respectively. The x-axis represents the physical position of the chromosomes. The y-axis represents 100 kb window-based *F_ST_*. The red dashed line represents the threshold (0.25) for high genetic divergence.

To clarify the genetic differentiation between northern and southern japonica, the *F_ST_* was calculated. The results showed that no highly genetically differentiated windows were found on Chr3, Chr7, Chr11, and Chr12 (Figure 4A–D). However, highly genetically differentiated windows existed on Chr1, Chr2, Chr4, Chr5, Chr6, Chr8, Chr9, and Chr10, with Chr5 showing the largest number of highly genetically differentiated windows in both NJRVs and SJRVs (Supplementary Table S2). Due to the notable variations in grain characteristics between NJRVs and SJRVs, we sought to identify the major genes that regulate rice grain traits and identified *RGA1* (*LOC_Os05g26890*) [34] and *SDF5* (*LOC_Os05g34325*), which were located in a region exhibiting significant genetic divergence on Chr5 (Figure 4E–L).

## 4. Discussion

### 4.1. Genetic Diversity Analyses between SJRVs and NJRVs

A previous study on the quality of 38 northern japonica and 15 southern japonica showed no significant differences in grain length and aspect ratio between southern japonica and northern japonica [35], but subsequent research in this study showed that northern japonica had more elongated grain length [10]. Ma et al. analyzed the quality of 5 southern japonica and 10 northern japonica varieties and found no significant differences in grain length, width, and aspect ratio [1]. This study analyzed 90 SJRVs and 51 NJRVs. No significant difference was found in grain length (Figure 2C), but NJRVs had narrower, thinner grains with larger grain aspect ratio (Figure 2E–G), differing from past studies. This may stem from sample size, as larger samples better represent genetic diversity [36]. Apart from varying sample sizes, the aforementioned studies exclusively utilized high taste japonica rice varieties from both southern and northern rice regions, with taste value exceeding 80 points. Consequently, these findings were specific to this particular group of japonica rice. In contrast, our study contained not only premium and high taste varieties, such as Chuxianggeng1, Jindao106, Shendao47, Jindao108, Beigeng1705, Jigeng81, Longdao16, Shendao6, Wuyoudao3, Chugeng37, Nangeng46, Yinguang, and Nangeng9108, but also incorporated diverse japonica rice varieties or lines with varying yields, qualities, tastes, and resistance traits.

### 4.2. Genetic Divergence Analysis between SJRVs and NJRVs

Due to the significant ecological differences between southern and northern rice cultivation regions [37], coupled with distinct breeding strategies, the agronomic traits of japonica rice in the south and north have diverged. Northern japonica rice stands out for its superior quality [10]. *DEP1*, a key regulator of rice panicle architecture [38], significantly reinforces lodging resistance in erect panicle japonica rice varieties [28], while southern japonica rice exhibits robust heat tolerance [39]. The effective panicle number and seed setting rate are, respectively, crucial factors influencing the yield potential of northern and southern japonica rice [40]. Japonica rice in the north focuses more on yield and resistance [41], while southern japonica rice prioritizes taste and quality improvements [42]. This study revealed geographic differentiation between SJRVs and NJRVs (Figure 3), featuring significant genetic divergence windows on Chr1, Chr2, Chr4, Chr5, Chr6, Chr8, Chr9, and Chr10 (Figure 4E–L), It corroborated their genetic divergence, consistent with earlier phenotypic and genomic findings.

**Figure 5:**
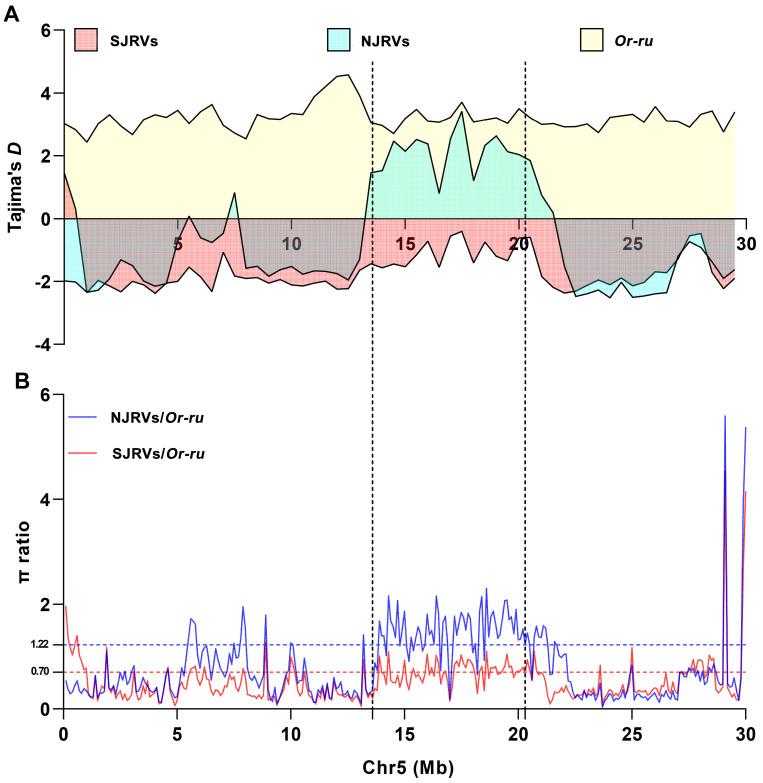
Neutral test analysis of SJRVs, NJRVs, and *Or-ru*. (**A**) Tajima’s *D* for SJRVs, NJRVs, and *Or-ru* on Chr5. The x-axis represents the physical position of the chromosomes. The y-axis represents 500 kb window-based Tajima’s *D* values. (**B**) Selection sweeps defined by100 kb window-based π ratios of NJRVs divided by *Or-ru* (blue line) and SJRVs divided by *Or-ru* (red line), respectively. Blue and red horizontal dashed line correspond to significance level of selection sweep for NJRVs/*Or-ru* (1.22) and SJRVs/*Or-ru* (0.70), respectively. The two black dotted lines represent the interval in which the windows of high genetic differentiation, i.e., Chr5: 13.6 Mb–21.1 Mb.

Multiple windows of high genetic differentiation were observed on Chr5 between southern and northern japonica rice. To investigate the possibility that the high genetic differentiation on Chr5 was affected by selection pressure, we obtained the sequencing data of 446 *Or-ru* from the OryzaGenome database (http://viewer.shigen.info/oryzagenome21detail/index.xhtml; accessed on 14 August 2021) and performed a neutral test with SJRVs and NJRVs. The results showed that SJRVs was under greater selection pressure at the windows of high genetic differentiation (Figure 5A) and had lower nucleotide diversity than NJRVs (Figure 5B).

The present study revealed that *RGA1* and *SFD5*, controlling grain characteristics [34,43], are located in a highly genetically differentiated window on Chr5. Given the notable disparities observed in grain width, aspect ratio, filled grain number, and grain number per panicle between NJRVs and SJRVs, it was postulated that the genes *RGA1* and *SFD5* played a role in mediating these phenotypic distinctions possibly. Owing to the considerable absence of SNPs within the coding sequences of the *RGA1* and *SFD5* genes in the sequencing data, a precise haplotype analysis of these two genes was rendered infeasible. Consequently, it is imperative to amplify all samples sequence of *RGA1* and *SFD5* in a subsequent phase, to enable a meticulous examination of haplotype variations. In addition, we considered analyzing differences in the expression levels of *RGA1* and *SFD5* between SJRVs and NJRVs.

## 5. Conclusions

SJRVs and NJRVs exhibited diversity differences and genetic differentiation. In addition, *RGA1* and *SFD5*, which control grain characteristics, were located in a highly genetically differentiated window on Chr5 between these two populations. Hence, it is suggested to conduct genetic introgression between SJRVs and NJRVs to broaden the genetic basis of local japonica rice germplasm. By exploiting their heterotic advantage, new japonica rice cultivars with superior comprehensive traits could be developed.

## Data Availability

All relevant data are available within the manuscript and its additional files. Sequence data that support the findings of this study have been deposited in the NCBI Sequencing Read Archive (SRA) database (BioProject ID: PRJNA848977; BioSample: SAMN29021483–SAMN29021632).

## Supplementary Materials

The following supporting information can be downloaded at: https://www.mdpi.com/article/10.3390/genes15091182/s1, Table S1: List of japonica rice varieties; Table S2: Highly genetically differentiated windows statistics.
