# Supplementary material for: Genetic Diversity and Divergence between Southern Japonica and Northern Japonica Rice Varieties in China
Source: Genes (Basel). 2024 Sep 9;15(9):1182. doi: 10.3390/genes15091182 (PMC11431492; doi:10.3390/genes15091182)
Supplement: Supplementary file 1 [file genes-15-01182-s001.zip › genes-3191577-supplementary.pdf]

Table S1. List of japonica rice varieties

Number	variety name	Province	Area	Number	variety name	Province	Area	Number	variety name	Province	Area
1	Chugeng37	Yunnan	Southern	48	Zhegeng23	Zhejiang	Southern	95	Tianfeng202	Liaoning	Northern
2	Chugeng40	Yunnan	Southern	49	Xiushui63	Zhejiang	Southern	96	Zi251	Liaoning	Northern
3	Chugeng28	Yunnan	Southern	50	Xiushui128	Zhejiang	Southern	97	YangengT65	Liaoning	Northern
4	Yinguang	Yunnan	Southern	51	Nonghu6	Zhejiang	Southern	98	Liaogeng399	Liaoning	Northern
5	Yungengnuo	Yunnan	Southern	52	Changnonggeng7	Zhejiang	Southern	99	Liaogeng1415	Liaoning	Northern
6	Chugeng7	Yunnan	Southern	53	Xiushui13	Zhejiang	Southern	100	Jindao108	Liaoning	Northern
7	Chugeng24	Yunnan	Southern	54	Ning88	Zhejiang	Southern	101	Tiegeng110	Liaoning	Northern
8	Chugeng26	Yunnan	Southern	55	Hangtian18	Zhejiang	Southern	102	Liaogeng212	Liaoning	Northern
9	Chugeng29	Yunnan	Southern	56	Yonggeng29	Zhejiang	Southern	103	Tiegeng20	Liaoning	Northern
10	Chugeng32	Yunnan	Southern	57	Xiushui620	Zhejiang	Southern	104	Tiegeng7	Liaoning	Northern
11	Chugeng39	Yunnan	Southern	58	Yongnuo34	Zhejiang	Southern	105	Jindao104	Liaoning	Northern
12	Tenggeng2	Yunnan	Southern	59	Jiawan12	Shanghai	Southern	106	Jindao106	Liaoning	Northern
13	Tenggeng4	Yunnan	Southern	60	Guangminggeng1	Shanghai	Southern	107	Shixindao1504	Liaoning	Northern
14	Yougeng18	Yunnan	Southern	61	Guangminggeng3	Shanghai	Southern	108	Yj07	Liaoning	Northern
15	Yougeng22	Yunnan	Southern	62	Guangminggeng2	Shanghai	Southern	109	Kaigeng1823	Liaoning	Northern
16	Hexi22-2	Yunnan	Southern	63	Hugeng1	Shanghai	Southern	110	Fuhedao255	Liaoning	Northern
17	Chupin3	Yunnan	Southern	64	Hanfeng	Shanghai	Southern	111	Fuhe5	Liaoning	Northern
18	Yunfengdao	Yunnan	Southern	65	Qingjiao307	Shanghai	Southern	112	Hungeng3	Liaoning	Northern
19	Hexi27-2	Yunnan	Southern	66	Huxianggeng151	Shanghai	Southern	113	Shendao6	Liaoning	Northern
20	Tengxi138	Yunnan	Southern	67	Nangengdao5055	Jiangsu	Southern	114	Youyu208	Liaoning	Northern
21	Qiangeng1917	Guizhou	Southern	68	Nangengdao52	Jiangsu	Southern	115	Meifengdao251	Liaoning	Northern
22	Bigeng1708	Guizhou	Southern	69	Nangeng46	Jiangsu	Southern	116	Liaogeng1697	Liaoning	Northern
23	Hekegeng	Guizhou	Southern	70	Ninggeng7	Jiangsu	Southern	117	Shenggeng9	Liaoning	Northern
24	Bidao126	Guizhou	Southern	71	Ninggeng8	Jiangsu	Southern	118	Liaogeng1515	Liaoning	Northern
25	Bidao08	Guizhou	Southern	72	Nangeng6212	Jiangsu	Southern	119	Liaogeng401	Liaoning	Northern
26	Bigeng44	Guizhou	Southern	73	Nangeng9108	Jiangsu	Southern	120	ZSJDG	Liaoning	Northern
27	Gz1410	Guizhou	Southern	74	Wuyugeng3	Jiangsu	Southern	121	Honggeng4	Liaoning	Northern
28	Hn17Y28	Hubei	Southern	75	Zhendao99	Jiangsu	Southern	122	Tie1507	Liaoning	Northern

29	Hn17Dd120	Hubei	Southern	76	Sugeng45	Jiangsu	Southern	123	Beigeng1705	Liaoning	Northern
30	Hn17Dd188	Hubei	Southern	77	Ninggeng7	Jiangsu	Southern	124	Youxindao18	Liaoning	Northern
31	Hn17Y191	Hubei	Southern	78	Wuyungeng21	Jiangsu	Southern	125	Tiegeng1603	Liaoning	Northern
32	Hn17Dd348	Hubei	Southern	79	Yanggeng687	Jiangsu	Southern	126	Shendao47	Liaoning	Northern
33	Hangeng10	Hubei	Southern	80	Xudao4	Jiangsu	Southern	127	Meifengdao271	Liaoning	Northern
34	Wangeng22	Anhui	Southern	81	Geng7623	Jiangsu	Southern	128	Meifengdao669	Liaoning	Northern
35	Tiangeng2	Anhui	Southern	82	Huaxi6	Jiangsu	Southern	129	Jigeng81	Jilin	Northern
36	Anxuanwan1	Anhui	Southern	83	Changnonggeng10	Jiangsu	Southern	130	Jigeng88	Jilin	Northern
37	Wandao68	Anhui	Southern	84	Zhendao11	Jiangsu	Southern	131	Jigeng812	Jilin	Northern
38	Dangyugeng2	Anhui	Southern	85	Ninggeng1	Jiangsu	Southern	132	Jigeng528	Jilin	Northern
39	Wandao36	Anhui	Southern	86	Ninggeng4	Jiangsu	Southern	133	Wuyoudao3	Heilongjiang	Northern
40	Wanhui9	Anhui	Southern	87	Wuyungeng31	Jiangsu	Southern	134	Longdao16	Heilongjiang	Northern
41	Yonggeng18	Zhejiang	Southern	88	Wuyungeng80	Jiangsu	Southern	135	Longgeng31	Heilongjiang	Northern
42	Zhegeng27	Zhejiang	Southern	89	Yzj5	Jiangsu	Southern	136	Kendao10	Heilongjiang	Northern
43	Chunjiang26	Zhejiang	Southern	90	Yanggengdao226	Jiangsu	Southern	137	Longyang11	Heilongjiang	Northern
44	Xiushui134	Zhejiang	Southern	91	Tianyushengdao	Shandong	Northern	138	Chuxianggeng1	Heilongjiang	Northern
45	Xiushui04	Zhejiang	Southern	92	Linhan1	Shandong	Northern	139	Songgeng65	Heilongjiang	Northern
46	Xiushui08	Zhejiang	Southern	93	Jingeng818	Tianjin	Northern	140	Longkedao868	Heilongjiang	Northern
47	Chunjiang15	Zhejiang	Southern	94	Jinyuan89	Tianjin	Northern	141	Dongnong601	Heilongjiang	Northern

Table S2. Highly genetically differentiated windows statistics

Serials	Chr	Start	End	F_{ST}	Serials	Chr	Start	End	F_{ST}
1	1	1400001	1500000	0.287345	45	5	19800001	19900000	0.337826
2	1	3000001	3100000	0.27392	46	5	19900001	20000000	0.448933
3	1	3300001	3400000	0.26981	47	5	20000001	20100000	0.432773
4	2	33500001	33600000	0.32607	48	5	20100001	20200000	0.574879
5	2	33600001	33700000	0.349341	49	5	20200001	20300000	0.482413
6	2	33700001	33800000	0.377332	50	5	20300001	20400000	0.356612
7	2	33800001	33900000	0.340618	51	5	20400001	20500000	0.452723
8	2	33900001	34000000	0.277399	52	5	20600001	20700000	0.364637
9	2	34000001	34100000	0.262683	53	5	20700001	20800000	0.412618
10	2	34200001	34300000	0.368276	54	5	20800001	20900000	0.314036
11	2	34300001	34400000	0.352274	55	5	20900001	21000000	0.325348
12	4	28400001	28500000	0.547826	56	5	21000001	21100000	0.323527
13	5	13600001	13700000	0.259166	57	6	9000001	9100000	0.286746
14	5	14300001	14400000	0.267976	58	6	9200001	9300000	0.331477
15	5	14400001	14500000	0.355058	59	6	9300001	9400000	0.320212
16	5	14600001	14700000	0.30684	60	6	9500001	9600000	0.299473
17	5	14700001	14800000	0.301881	61	8	25600001	25700000	0.3373
18	5	15000001	15100000	0.315709	62	8	26100001	26200000	0.339443
19	5	15100001	15200000	0.282125	63	8	26200001	26300000	0.263453
20	5	15500001	15600000	0.294603	64	8	26300001	26400000	0.296551
21	5	15600001	15700000	0.302749	65	8	26400001	26500000	0.303638
22	5	15700001	15800000	0.314274	66	8	26500001	26600000	0.44819
23	5	16000001	16100000	0.293508	67	8	26700001	26800000	0.317539
24	5	16200001	16300000	0.33731	68	8	27300001	27400000	0.267167
25	5	16300001	16400000	0.256153	69	9	15000001	15100000	0.255823
26	5	16700001	16800000	0.254238	70	9	17100001	17200000	0.259101
27	5	17000001	17100000	0.29309	71	10	15100001	15200000	0.312165
28	5	17100001	17200000	0.280586	72	10	15200001	15300000	0.277591
29	5	17300001	17400000	0.32479	73	10	15300001	15400000	0.325265
30	5	17400001	17500000	0.253525	74	10	15400001	15500000	0.257352
31	5	17500001	17600000	0.370069	75	10	15600001	15700000	0.278916
32	5	17600001	17700000	0.336622	76	10	15800001	15900000	0.303367
33	5	17800001	17900000	0.330482	77	10	15900001	16000000	0.409598
34	5	18200001	18300000	0.268643	78	10	16000001	16100000	0.384118
35	5	18500001	18600000	0.28396	79	10	16100001	16200000	0.32525
36	5	18700001	18800000	0.350695	80	10	16200001	16300000	0.329693
37	5	18800001	18900000	0.369766	81	10	16400001	16500000	0.296769
38	5	18900001	19000000	0.261992	82	10	16600001	16700000	0.364154
39	5	19000001	19100000	0.274018	83	10	16700001	16800000	0.361377
40	5	19100001	19200000	0.274651	84	10	16800001	16900000	0.303458
41	5	19200001	19300000	0.278862	85	10	17400001	17500000	0.261884
42	5	19300001	19400000	0.339468	86	10	17500001	17600000	0.301804
43	5	19400001	19500000	0.41437	87	10	17600001	17700000	0.294349
44	5	19600001	19700000	0.261915	88	10	17700001	17800000	0.29699
